# Polycondensed Peptide-Based Polymers for Targeted Delivery of Anti-Angiogenic siRNA to Treat Endometriosis

**DOI:** 10.3390/ijms25010013

**Published:** 2023-12-19

**Authors:** Anna Egorova, Marianna Maretina, Iuliia Krylova, Anton Kiselev

**Affiliations:** 1 Laboratory of Molecular Genetics and Gene Therapy, D.O. Ott Research Institute of Obstetrics, Gynecology and Reproductology, Mendeleevskaya Line 3, 199034 Saint-Petersburg, Russia; egorova_anna@yahoo.com (A.E.); marianna0204@gmail.com (M.M.); 2 Department of Pathology, Pavlov First Saint-Petersburg State Medical University, L’va Tolstogo Street 6-8, 197022 Saint-Petersburg, Russia; emerald2008@mail.ru

**Keywords:** endometriosis, siRNA delivery, peptide-based carriers, gene therapy, VEGFA, integrins, cross-linking peptide, anti-angiogenic therapy

## Abstract

Endometriosis (EM) is a prevalent gynecological disease characterized by the abnormal growth of tissue similar to the endometrium outside of the uterus. This condition is accompanied by the development of new blood vessels in endometriotic lesions. While surgical intervention is effective in removing endometriotic lesions, some patients require multiple surgeries. Therefore, finding non-surgical treatments for EM is of great interest. One of the promising approaches is anti-angiogenic therapy using siRNA-therapeutics to target the expression of the VEGFA gene. Peptide-based polymers have shown promise as siRNA delivery systems due to their biocompatibility and ease of modification. We conducted a study to evaluate the effectiveness of the R6p-cRGD peptide carrier as a non-viral vehicle for delivering siRNA to endothelial cells in vitro and endometrial implants in vivo. We investigated the physicochemical properties of the siRNA-complexes, assessed cellular toxicity, and examined the efficiency of GFP and VEGFA genes silencing. Furthermore, we tested the anti-angiogenic effects of these complexes in cellular and animal models. The transfection with siRNA complexes led to a significant increase in VEGFA gene knockdown efficiency and a decrease in the migration of endothelial cells. For the animal model, we induced endometriosis in rats by transplanting endometrial tissue subcutaneously. We evaluated the efficiency of anti-angiogenic therapy for EM in vivo using anti-VEGF siRNA/R6p-RGD complexes. During this assessment, we measured the volume of the implants, analyzed VEGFA gene expression, and conducted CD34 immunohistochemical staining. The results showed a significant decrease in the growth of endometriotic implants and in VEGFA gene expression. Overall, our findings demonstrate the potential of the R6p-cRGD peptide carrier as a delivery system for anti-angiogenic therapy of EM.

## 1. Introduction

Therapeutic anti-angiogenesis aims to halt the growth of blood vessels in tumors and other tissues that rely on angiogenesis for growth. This can be achieved by blocking the production or functioning of pro-angiogenic factors, e.g., vascular endothelial growth factor A (VEGFA) that plays an important role in angiogenesis and vascular permeability [1]. VEGFA is upregulated in response to tissue hypoxia, or low oxygen levels that are common at early stages of tumors development. By impeding the formation of blood vessels, the growth and spread of diseased tissue can be suppressed. Potential targets for antiangiogenic therapy include substances that stimulate the development of new vessels, receptors for them, as well as the endothelial cells of the newly formed vessels. Considering anti-angiogenic therapy as a potential strategy for effectively treating certain patient populations, such as those affected by tumors, as well as other conditions characterized by pathological angiogenesis, like age-related macular degeneration or endometriosis, would prove highly beneficial [2,3]. Numerous applications of anti-angiogenic therapy have been reported to date, particularly in the treatment of solid tumors [1]. Currently, anti-angiogenic drugs are available in various forms including small-molecule multitargeted inhibitors, as well as highly specific macromolecules such as antibodies and small interfering RNA (siRNA) [4].

Endometriosis (EM) is a well-known inflammatory disease that primarily affects women of reproductive age and is influenced by estrogen levels. It is characterized by the presence of endometrial tissue outside the uterus [5]. The primary methods for treating EM predominantly revolve around surgical procedures and hormonal therapies; unfortunately, these approaches often come with a range of negative side effects, such as high recurrence rate and the requirement of multiple surgeries, along with dependence on the long-term administration of hormonal drugs [5,6,7]. While the exact cause of endometriosis remains largely unknown, researchers have put forward several theories, including retrograde menstruation, coelomic metaplasia, and lymphatic and vascular metastasis [6]. These theories suggest various potential sources of endometriotic lesions. However, what appears to be crucial in the progression of EM is the promotion of enhanced angiogenesis. The maintenance and development of endometriosis depend greatly on the stimulation of new blood vessel formation, thus the application of therapeutic anti-angiogenesis can effectively alleviate the symptoms of the disease [3,4]. VEGF blockers and inhibitors have shown significant potential in animal models by effectively reducing the number of endometriotic implants, decreasing vascular density, promoting apoptosis, and decreasing VEGF levels in peritoneal fluid [8,9,10]. The intravenous application of monoclonal anti-VEGF antibody in EM therapy has yielded promising results in a human clinical trial [11]. However, undesirable side effects may arise when anti-angiogenic drugs are used throughout the body, such as the unintentional impact on fertility caused by the non-specific targeting of the normal endometrial tissue [12,13]. Thus, targeted drug delivery is crucial for the effective treatment of EM while preserving fertility.

Recent advancements in oligonucleotide drug research have paved the way for the development of RNAi-based anti-angiogenic therapeutics. These siRNA molecules can be effectively encapsulated into lipid- or polymer-based nanoparticles (NPs), which are further enhanced with ligands to ensure targeted delivery through receptor-mediated mechanisms. When undergoing EM treatment, it is important to direct the delivery of anti-angiogenic siRNA to the endothelium of newly formed vessels of endometriotic lesions, as this is the intended target tissue. To achieve this goal a proper ligand-receptor pair should be chosen to avoid non-specific delivery. Previously, promising results have been obtained for NPs modified by various types of RGD motive, a ligand which is well-known for ανβ3 integrin binding [14,15]. The role of this receptor is crucial in the development of endometrial receptivity and new blood vessels formation in the EM pathogenesis [16,17]. Notably, the neoangiogenic processes observed in EM exhibit similar markers to those found in tumor neoangiogenesis [17,18]. Furthermore, there is an overexpression of ανβ3 integrin in the endothelial cells of ectopic endometrium in EM patients. Previous studies have reported a down-regulated expression of ανβ3 integrin in the eutopic endometrium of women with EM compared to fertile individuals [19]. Thus, targeting ανβ3 integrin with anti-angiogenic drugs may potentially protect the normal endometrium in endometriosis patients.

The use of peptide-based siRNA carriers offers numerous advantages compared to other polymer-based delivery systems. These include biocompatibility, biodegradability, minimal toxicity, and the ability to easily modify them using a modular approach [20]. Peptide-based vehicles are currently being developed to address the limitations of viral vectors (e.g., high cost, low packaging capacity, poor tissue selectivity, risk of liver toxicity, and immunogenicity) and to create artificial virus-mimicking systems, despite their lower transfection efficiency [21,22]. Peptide carriers enriched with arginine, which are part of the cationic cell penetrating peptides (CPPs) class, have proven to be highly successful in delivering genes both in vitro and in vivo [23]. Previous research has demonstrated that CPPs that are crosslinked via disulfide bonds significantly enhance transfection while maintaining low levels of cytotoxicity. Disulfide bonds can be easily reduced by glutathione, a common reductive agent found in the cytoplasm. This, in turn, enables the release of the cargo [24].

Previously, we created peptide NPs that were modified with different types of ανβ3 integrin ligand—iRGD and cRGD [25,26,27,28]. The iRGD-modified nanoparticles combine two peptides, the ligand-bearing RGD1, and the crosslinking R6, to form RGD1-R6 polyplexes. These polyplexes are formed by non-covalently mixing the peptides with nucleic acids, while also simultaneously forming disulfide links between R6 peptides [26]. An alternate delivery system, R6p-cRGD, was designed on the basis of polycondensed R6p polymer modified with cyclic RGD ligand [27]. Developed NPs proved to be highly effective and specific in gene delivery to ανβ3 integrin-expressing cells [26,27]. Additionally, using iRGD-modified NPs, we successfully down-regulated VEGFA gene expression and demonstrated anti-angiogenic effects in vitro. Further anti-angiogenic action of iRGD-modified anti-VEGFA siRNA/RGD1-R6 polyplexes was confirmed in vivo using an established EM rat model [29].

This study aims to assess the potential of R6p-cRGD, a polycondensed peptide-based polymer, as a siRNA delivery system in the treatment of EM by targeting angiogenesis. The siRNA-polyplexes were assessed for their transfectional and toxic characteristics in both cancer and endothelial cells expressing ανβ3 integrin. Additionally, the therapeutic effects of the polyplexes were studied in a rat model of EM. The model was induced via the autotransplantation of uterine horn fragments onto the outer peritoneal surface and the implants were transfected by anti-VEGFA siRNA bearing polyplexes.

## 2. Results and Discussion

Cationic polymers are widely used as non-viral gene delivery vectors due to their ability to form nanoscaled polyplexes with nucleic acids through electrostatic interactions. However, a challenge arises with the conflicting stabilities of the polyplexes in different environments—extracellular and intracellular. The solution to this stability paradox lies in leveraging the high redox potential inside cells by incorporating bioreducible disulfide moieties, as was demonstrated in previous studies [30,31].

In this study we evaluated the potential of an R6p-cRGD carrier obtained via oxidative polycondensation of arginine-histidine-rich cysteine-flanked R6 peptides with the inclusion of cyclic CRGDy ligand moiety in the reaction mixture. The resulting polymers consist of up to 18–19 monomer units of R6 peptide and are flanked by RGD-ligand moiety which acts as a chain breaker during polycondensation. This type of carrier was successfully applied in plasmid DNA delivery to ανβ3 integrin-expressing cells [27].

### 2.1. siRNA-Binding and siRNA Protection Properties of the Carriers

**Figure 1:**
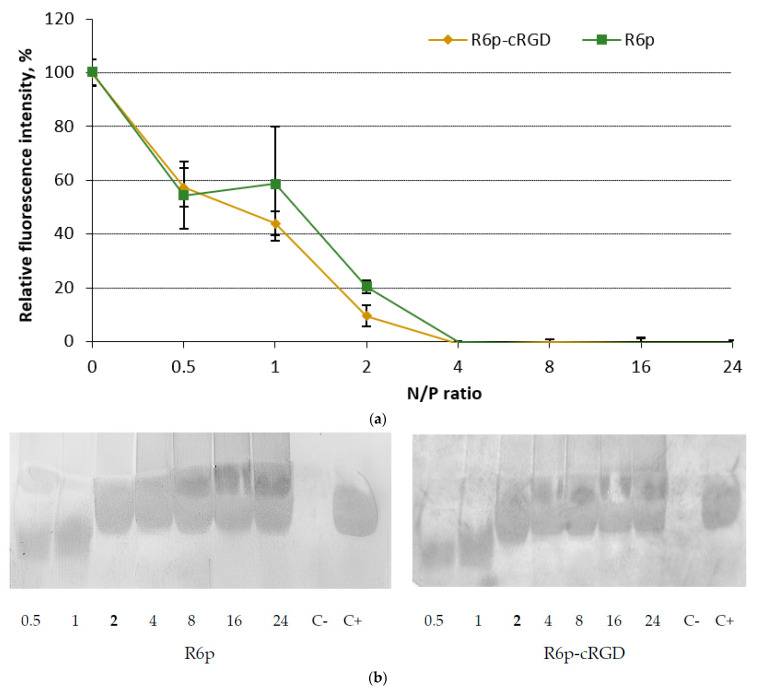
Evaluation of siRNA-binding and siRNA protection properties in complexes with R6p and R6p-cRGD carriers: (**a**) Sybr Green displacement assay of siRNA. Values are the mean ± S.D. of *n* = 9 individual samples from three independent experiments; statistical significance was assessed by ordinary one-way ANOVA; (**b**) RNase A protection assay. N/P ratio in bold indicates full siRNA protection. Controls: C− is ‘naked’ siRNA treated with RNase A; C+ is untreated siRNA.

To evaluate the binding efficiency of siRNA with the polycondensed carriers, we conducted a Sybr Green displacement assay (Figure 1a). As the concentration of the carriers increased, we observed a gradual rise in the density of the complexes. Remarkably, the fluorescence intensity of the peptide/siRNA complexes exhibited a sharp decline at an N/P ratio of two, reaching a significant reduction of 9–20%. When the N/P ratio reached four, the fluorescence intensity dropped to zero. These findings further emphasize the strong interaction between siRNA and the polycondensed carriers.

The capacity of carriers to bind to siRNA directly has a significant impact on their ability to protect against nuclease degradation. To evaluate the integrity of the siRNA in the complexes, we conducted an RNase A protection assay. Our analysis revealed that the polycondensed carriers we studied were able to effectively shield siRNA from nuclease degradation at an N/P ratio of 2 (Figure 1b). This is correlated with a notable decrease in Sybr Green fluorescence intensity, as determined by the siRNA binding assay (Figure 1a). It is worth noting the mechanical properties of double-stranded RNA, specifically its persistence length, is approximately 70 nm. This corresponds to siRNA molecules that are 185 and 260 base pairs in length [32]. Thus, short siRNA molecules cannot be condensed by polycations in the classical manner; however, their electrostatic interaction can result in molecular collapse and the formation of nuclease-resistant siRNA-complexes [33]. In summary, the data we have obtained clearly demonstrate the remarkable capacity of the R6p-cRGD carrier to bind to siRNA and provide it with protection.

**Figure 2:**
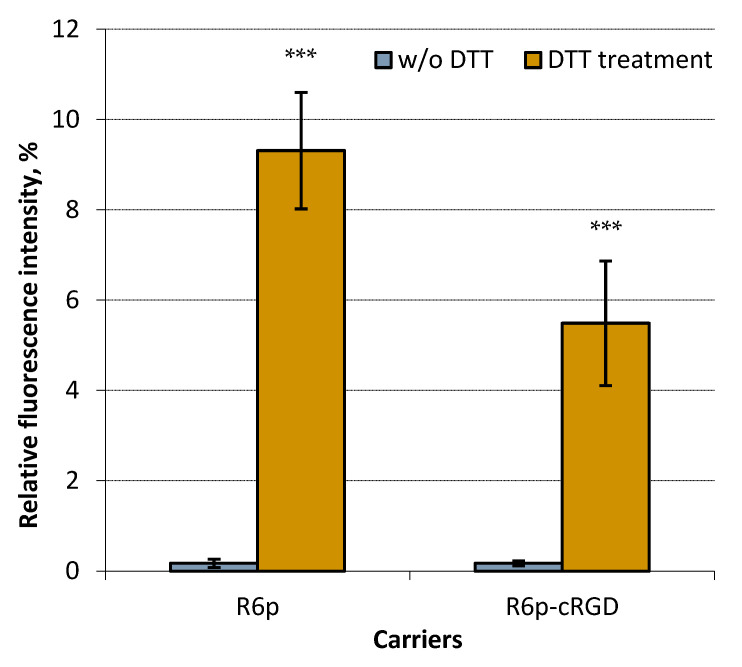
siRNA release after DTT treatment of siRNA-complexes with R6p and R6p-cRGD carriers formed at N/P ratio 8/1. Values are the mean ± S.D. of *n* = 9 individual samples from three independent experiments; statistical significance was assessed by ordinary one-way ANOVA. ***—*p* < 0.001 compared to untreated complexes.

In order to further examine the siRNA binding properties of the polycondensed carriers, we investigated the influence of disulfide bonds on the formation of complexes. It is well known that reducible polycations, such as the ones used in this study, are susceptible to destabilization by dithiothreitol (DTT) [34]. Therefore, the complexes were treated with DTT and the extent of siRNA release was assessed. Treatment with DTT was carried out on siRNA-polyplexes at an N/P ratio of 8/1, which has been shown in previous data to result in complete siRNA binding and protection. As depicted in Figure 2, incubating the complexes with DTT caused a significant increase in Sybr Green fluorescence because of partial release of siRNA. Consequently, the results of the DTT treatment corroborate the crucial role of disulfide cross-linking in enhancing siRNA binding, as previously demonstrated for DNA complexes formed using oxidatively condensed polypeptides [27,35].

### 2.2. Size and ʐ-Potential of siRNA-Complexes

Polyplex size plays a crucial role in determining how they interact with cells and how long they stay in circulation [36]. Previous studies have shown that NPs with sizes below 100 nm can enter cells through caveolin-mediated endocytosis, while larger complexes ranging from 100–200 nm can utilize clathrin-mediated endocytosis [37]. Additionally, NPs with sizes in the range of 70–200 nm exhibit the optimal combination of prolonged bloodstream circulation and effective tumor penetration [38]. These findings emphasize the importance of carefully considering polyplex size as a key parameter in biomedical applications.

The size and ʐ potential of the siRNA-complexes were determined at N/P ratios of 8/1 and 16/1. These charge ratios ensure that the complexes remain colloidally stable and effectively bind and protect the siRNA. We observed that the size of the complexes ranged between 100 and 200 nm (Table 1). The polydispersity index (PDI) value, which reflects their size distribution quality, was consistently around 0.2 for all N/P ratios. A PDI value of less than 0.2 indicates that the complexes have a uniform size distribution. This optimal size range makes it possible for the complexes to penetrate the cell membrane through clathrin-mediated endocytosis. This mechanism has previously proven to be the most probable pathway for the cellular uptake of cRGD ligand-modified NPs [39].

The siRNA complexes were analyzed for their zeta-potential at the same charge ratios. As anticipated, all the polyplexes exhibited a positive charge on their surface, with values ranging from +23 to +32 mV (Table 1). This positive charge could potentially enhance cellular uptake and consequently facilitate the down regulation of gene expression.

The characterization of R6p-cRGD-siRNA-polyplexes was compared to recent data on the physico-chemical properties of RGD1-R6-siRNA-polyplexes, which are composed of matrix-polymerized R6 oligomers and non-covalently bound iRGD-modified RGD1 peptide [29]. Despite both types of polyplexes consisting of the R6 cross-linking peptide, we observed notable differences between them. At the same charge ratios, RGD1-R6-siRNA-polyplexes at N/P ratios of 8/1 and 16/1 exhibited larger sizes (350.9 ± 3.6 nm and 229.2 ± 4.4 nm, respectively) and lower charges (13.1 ± 0.31 mV and 18.8 ± 0.54 mV, respectively) compared to R6p-cRGD-siRNA-polyplexes. This discovery may indicate a distinction between the two types of R6 peptide polymerization, leading to the synthesis of polypeptides with varying lengths. It can be hypothesized that polycondensed R6p polymers possess a superior binding capacity than R6-based oligomers, demonstrating a higher charge density that facilitates the formation of more positively charged complexes. This explanation is supported by previous findings on the comparison of peptide-based polymers formed by oxidative polycondensation and matrix polymerization [31,35].

### 2.3. Assessment of Cytotoxicity of siRNA-Complexes

Two cell lines were selected for cellular experiments, specifically because they express ανβ3 integrins on their surface [40,41]. The MDA-MB 231 cell line, which stably expresses the GFP reporter gene, was used to initially examine the biological properties of the polyplexes. On the other hand, the hybridoma EA.hy926 replicates the primary morphological, phenotypical, and functional characteristics of the human endothelium [42]. The endothelium is suggested as a target tissue for the polyplexes and was used for in vitro assessement of their therapeutic potential.

**Figure 3:**
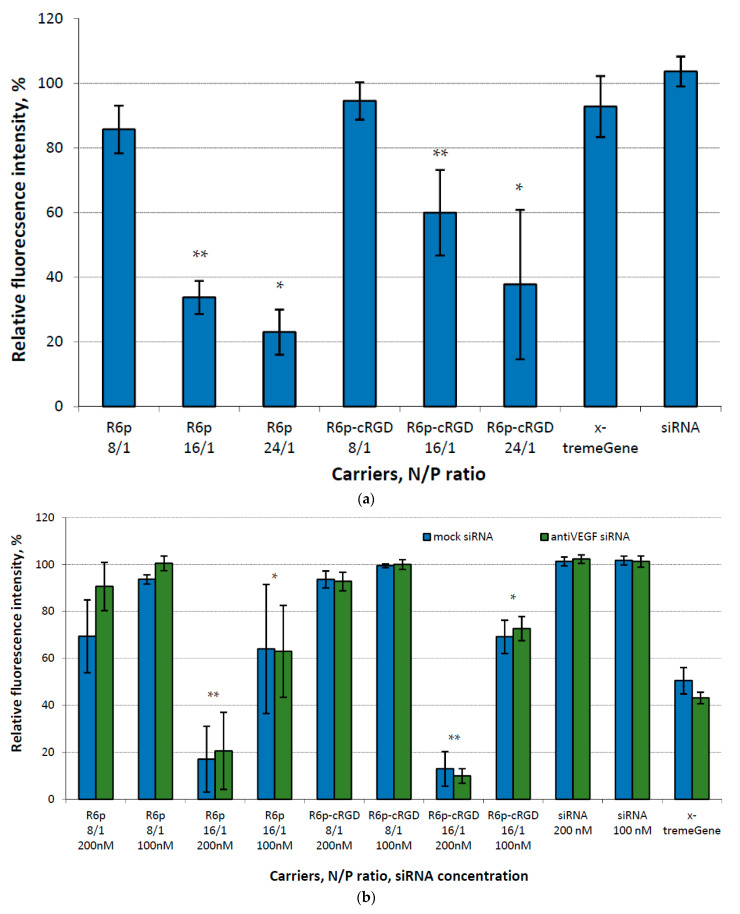
Cytotoxicity evaluation of siRNA-complexes at different N/P ratios in (**a**) MDA-MB 231 cells at siRNA concentration 200 nM and (**b**) EA.hy926 cells at siRNA concentrations 200 nM and 100 nM. Values are the mean ± S.D. of *n* = 9 individual samples from three independent experiments; statistical significance was assessed by ordinary one-way ANOVA (**a**) and two-way ANOVA (**b**). *—*p* <0.05, **—*p* <0.01 compared to corresponding complexes formed at N/P ratio 8/1.

Given the critical role of cytotoxicity in determining the potential application of the studied carriers as siRNA delivery systems, the polyplexes were thoroughly evaluated for their cytotoxic properties in both cell lines. This examination was conducted at N/P ratios of 8/1, 16/1, and 24/1. Naked siRNA was used as a negative control, and siRNA/x-tremeGene complexes were used as a positive control. For the initial experiments, we used a relatively high siRNA concentration (200 nM) and mock siRNA as a cargo for the polyplexes. The data presented in Figure 3a show significant differences in toxicity between polyplexes formed at higher N/P ratios and those formed at an 8/1 charge ratio in MDA-MB 231 cells. We discovered that polyplexes with charge ratios of 16/1 and 24/1 demonstrated a remarkably high level of cytotoxicity. This finding raises concerns about their suitability for future applications. In order to address this issue, we made modifications to the cytotoxicity testing in the EA.hy926 cell line. Specifically, we incorporated samples with a lower concentration of siRNA (100 nM) and introduced anti-VEGFA siRNA to examine the potential harm that VEGFA gene silencing could impose on endothelial cells. Additionally, polyplexes formed at a 24/1 charge ratio were excluded from the testing. Based on the data we obtained, clear evidence indicates that polyplexes with higher N/P ratios, tested at a concentration of 200 nM, display pronounced cytotoxicity towards endothelial cells. Similarly, x-tremeGene-based complexes also exhibited cytotoxicity towards to EA.hy926 cells in contrast to MDA-MB 231 cells. VEGFA expression knockdown cannot be a reason of the cytotoxicity because no difference is found between anti-VEGFA and mock siRNA transfection. It can be supposed that EA.hy926 hybridoma cells are more sensitive to non-viral transfection at given siRNA concentration in comparison to secondary MDA-MB 231 cell line. On the other hand, polyplexes formed at a charge ratio of 16/1 and tested at a lower concentration of siRNA showed a lesser degree of toxicity. Nevertheless, it is worth noting that all polyplexes at an 8/1 charge ratio were found to be completely non-toxic (Figure 3b).

Based on the results of the cytotoxicity testing, we can conclude that the addition of cRGD to the carrier composition does not increase toxicity. This is supported by the fact that there was no difference in toxicity observed between the ligand-modified and control polyplexes. However, it is worth noting that the high net positive charge density of the polyplexes at high N/P ratios is likely a major contributor to the observed toxicity. Previous studies have shown that, when the zeta potential values exceed +30 mV, cellular toxicity can be induced by polyplexes [43].

### 2.4. Assessment of GFP and VEGFA Gene Expression Silencing by siRNA-Complexes

**Figure 4:**
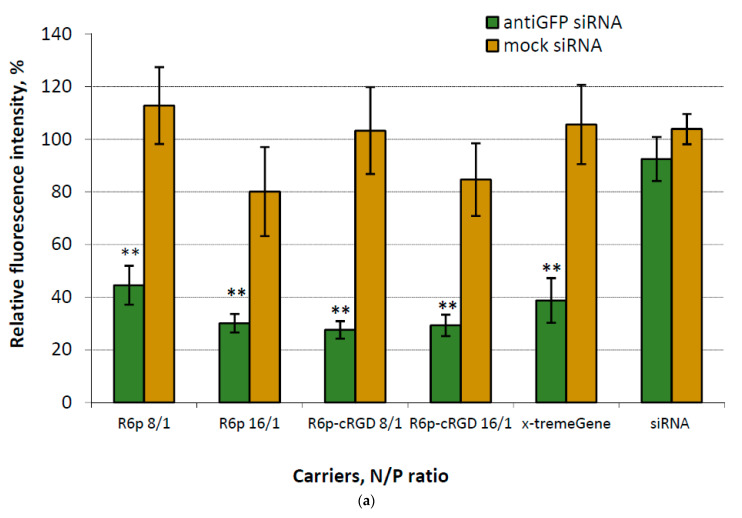

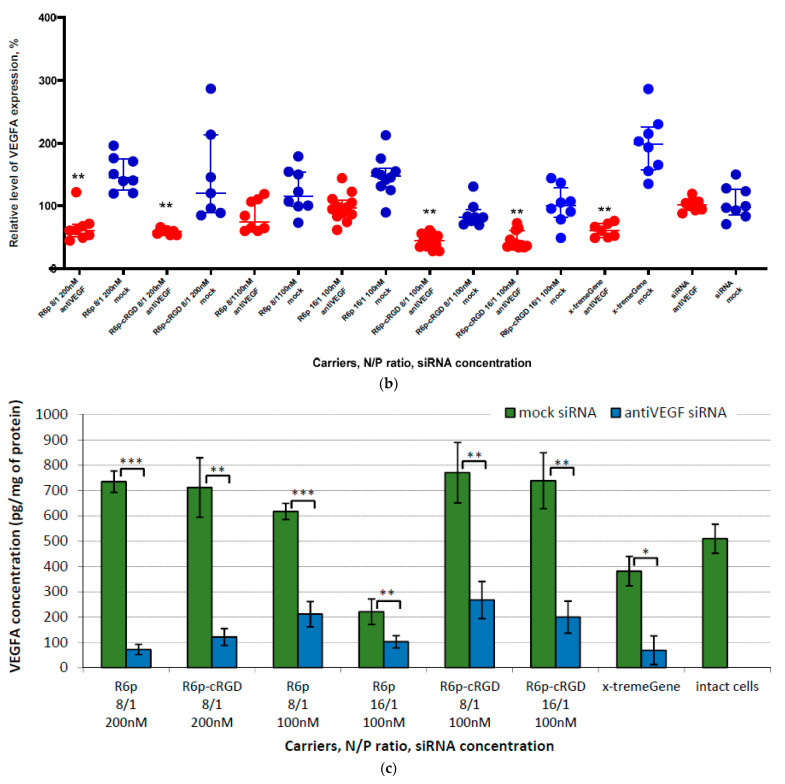
siRNA transfection efficacy evaluation: (**a**)—silencing of GFP expression after treatment of MDA-MB-231-GFP+ cells with the siRNA-complexes at siRNA concentration 200 nM. **—*p* < 0.01 when compared with negative control. The data are shown as the mean ± S.D. of *n* = 9 individual samples from three independent experiments; statistical significance was assessed by ordinary one-way ANOVA; (**b**)—silencing of VEGFA gene expression after treatment of E.A.Hy926 cells with the anti-VEGFA siRNA-complexes (red circles) and mock siRNA-complexes (blue circles) at different siRNA concentrations. **—*p* < 0.01 when compared with negative control. The data are shown as the median ± interquartile range of *n* = 8 individual samples from four independent experiments; statistical significance was assessed by ordinary two-way ANOVA; (**c**)—VEGFA protein production (pg/total protein) by EA.Hy926 cells after the transfection with siRNA-complexes. *—*p* < 0.05, **—*p* < 0.01, ***—*p* < 0.001 when compared with cells treated by corresponding mock siRNA-complexes. The data are shown as the mean ± S.D. of *n* = 9 individual samples from three independent experiments; statistical significance was assessed by ordinary two-way ANOVA.

In order to determine the transfection efficiency of siRNA/R6p and siRNA/R6p-cRGD polyplexes in αvβ3 integrin-positive MDA-MB 231-GFP+ cells, a corresponding siRNA was used along with mock siRNA to confirm RNAi-based silencing. The cells were treated with the polyplexes at N/P ratios of 8/1 and 16/1, whereas naked siRNA was used as a negative control and siRNA/x-tremeGene complexes as a positive control (Figure 4a). The fluorescence intensity of intact MDA-MB 231-GFP+ cells was taken to be 100%.

Figure 4a demonstrates that the delivery of mock siRNA was ineffective in suppressing the expression of the GFP gene. However, through the utilization of anti-GFP siRNA-complexes, we achieved a silencing efficiency ranging from 56% to 73%, as evidenced by the reduction in GFP fluorescence in MDA-MB-231 cells. This finding confirms that the suppression of GFP gene expression is indeed accomplished through a specific RNAi-based mechanism. The silencing efficacy of all studied siRNA-complexes was equivalent to that of control x-tremeGene-based polyplexes.

The effectiveness of transfection experiments was tested in endothelial hybridoma EA.Hy926 using two different concentrations of siRNA. The efficacy of the polyplexes was compared to the gene expression level achieved when naked siRNA was delivered, which is roughly equivalent to the baseline expression level of the VEGFA gene (100%). Our findings revealed that the control anti-VEGFA siRNA/R6p polyplexes were largely ineffective in downregulating the expression of the target gene. However, the complexes modified with cRGD-ligand showed a substantial twofold reduction in gene expression (Figure 4b). Additionally, we conducted an analysis of the VEGFA protein levels following the administration of anti-VEGFA siRNA/R6p-cRGD polyplexes in the endothelial cell culture. This analysis confirmed a substantial decrease in the production of VEGFA protein as a result of siRNA-induced gene silencing. It is worth noting that, despite the relatively low effectiveness of R6p-polyplexes in downregulating VEGFA gene expression, they demonstrated a comparable efficacy in reducing VEGFA protein production (Figure 4c). It is crucial to note that, when the VEGFA gene expression is reduced through RNA interference, there is a significant and enhanced reduction in VEGFA protein production. This can be attributed to the fact that a single VEGFA mRNA molecule can serve as a template for the translation of multiple VEGFA protein molecules. The destruction of these mRNA molecules by the RNAi mechanism leads to an amplified impact on the protein level, thereby guaranteeing the therapeutic efficacy of the administered siRNAs. However, the correlation between mRNA and protein abundances in the cell is not linear and multiple biological factors have been identified that influence this relationship [44].

In summary, our transfection studies affirm that the polyplexes we have developed successfully inhibit the expression of the target gene and the production of the corresponding protein.

### 2.5. Assessment of Endothelial Cells Migration and Proliferation Inhibition by siRNA-Complexes

In general, the active processes in angiogenesis, which involve the formation of new blood vessels, are primarily observed during the development of embryos. Once an organism is fully formed, the requirement for angiogenesis is limited to specific situations such as tissue repair, fetal development, and the female reproductive cycle [45]. On the contrary, the progression of numerous human diseases hinges on the intensity of the neoangiogenesis process, which involves the formation of a fresh network of capillaries, and thus depends on the proliferation and migration of endothelial cells [46]. Neoangiogenesis is the process in which endothelial cells rapidly multiply and form intricate three-dimensional structures. This leads to the creation of a network of blood vessels [47]. The rate at which anti-angiogenic siRNA-bearing polyplexes inhibit the migration and proliferation of endothelial cells is a crucial functional characteristic which was assessed in this study via different methods.

**Figure 5:**
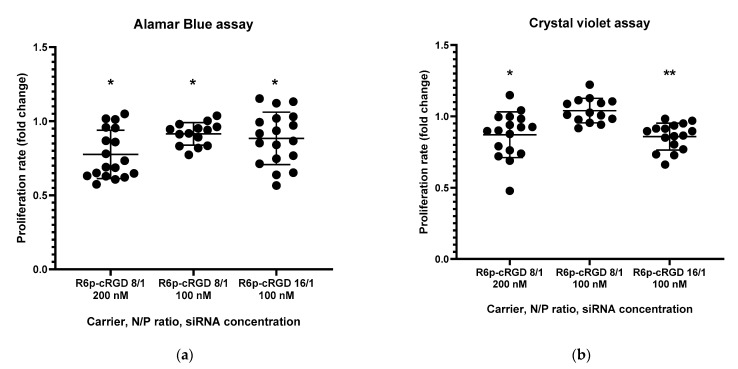
Fold change in proliferation rate of EA.Hy926 cells after treatment with anti-VEGFA siRNA/R6p-cRGD polyplexes analyzed by Alamar blue assay (**a**) and by crystal violet assay (**b**); *—*p* < 0.05, **—*p* < 0.01 when compared with cells treated by mock siRNA/R6p-cRGD polyplexes. The data are shown as the mean ± S.D. of *n* = 15 individual samples from five independent experiments; statistical significance was assessed by ordinary one-way ANOVA.

The Alamar blue assay and crystal violet assay were employed to investigate the impact of anti-VEGFA siRNA delivery on endothelial cell proliferation, utilizing the R6p-cRGD carrier (Figure 5). To establish a negative control, mock siRNA-polyplexes were employed. Endothelial cells were transfected with siRNA/R6p-cRGD polyplexes created at charge ratios of 8/1 and 16/1, with an siRNA concentration of 100 nM for both N/P ratios. Additionally, the siRNA concentration of 200 nM was exclusively utilized for polyplexes at a 16/1 ratio.

Data presented in Figure 5 reveal a noteworthy decrease in the number of living endothelial cells following the administration of anti-VEGFA siRNA/R6p-cRGD polyplexes. The results obtained from various methods generally align, except for the sample with a low concentration of polyplexes formed at a charge ratio of 8/1, where no disparity in cell numbers was found between mock siRNA and anti-VEGF siRNA treatment. This observation suggests that a lower concentration of siRNA treatment may lead to a less pronounced inhibition of endothelial cell proliferation. However, an increase in the charge ratio of the polyplexes at the same siRNA concentration results in an amplified inhibitory effect.

**Figure 6:**
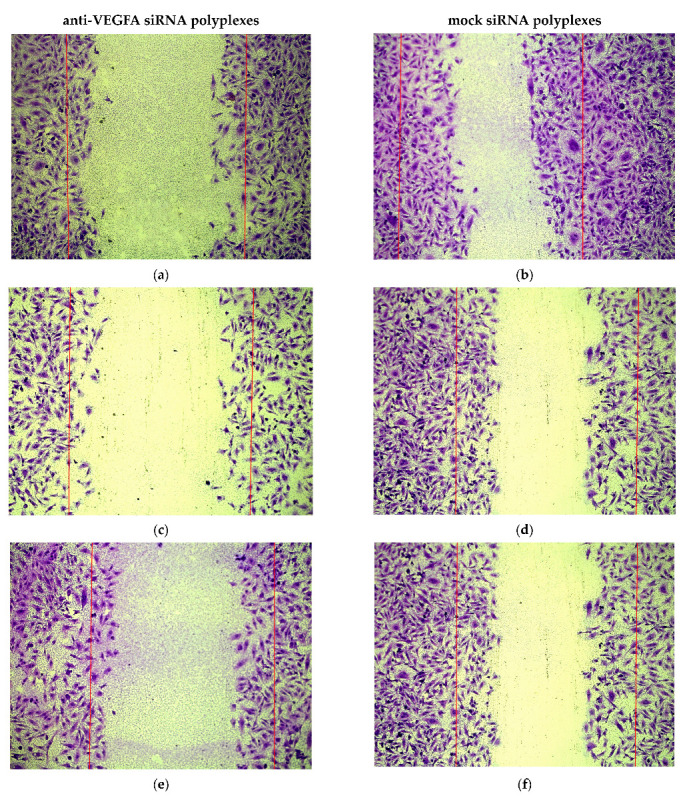

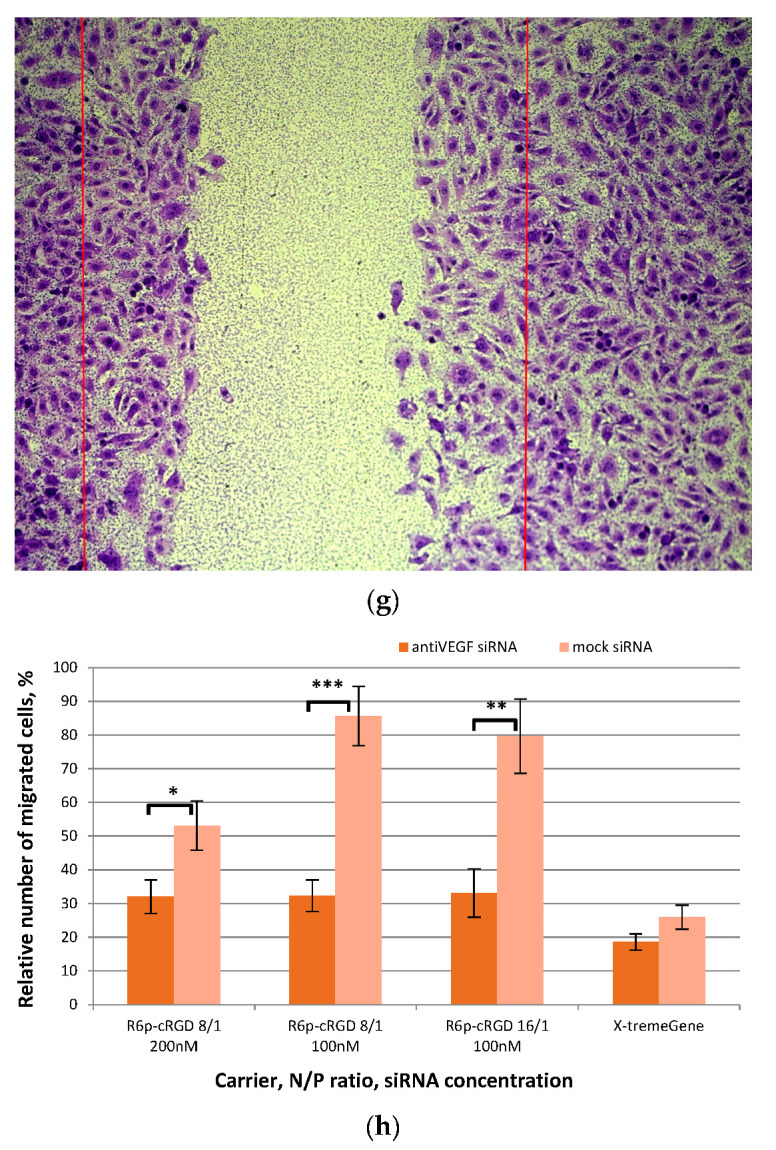
Visual appearance of migrated EA.Hy926 cells after treatment with (**a**) 100 nM of anti-VEGFA siRNA/R6p-cRGD at N/P ratio 8/1, (**b**) 100 nM of mock siRNA/R6p-cRGD at N/P ratio 8/1, (**c**) 200 nM of anti-VEGFA siRNA/R6p-cRGD at N/P ratio 8/1, (**d**) 200 nM of mock siRNA/R6p-cRGD at N/P ratio 16/1, (**e**) 100 nM of anti-VEGFA siRNA/R6p-cRGD at N/P ratio 16/1, (**f**) 100 nM of mock siRNA/R6p-cRGD at N/P ratio 16/1, (**g**) intact cells, (**h**) relative number of migrated EA.Hy926 cells after treatment with different concentrations of siRNA/R6p-cRGD polyplexes at N/P ratios 8/1 and 16/1. *—*p* < 0.05, **—*p* < 0.01, ***—*p* < 0.001 when compared with cells treated by mock siRNA-polyplexes. The data are shown as the mean ± S.D. of *n* = 15 individual samples from five independent experiments; statistical significance was assessed by ordinary one-way ANOVA.

We conducted a scratch migration test (Figure 6) to evaluate the impact of anti-VEGFA siRNA/R6p-cRGD-polyplexes treatment on the migration rate of EA.Hy926 cells. To measure quantitative changes, we analyzed images captured after the scratch-healing process in the endothelial monolayer. We employed N/P ratios of 8/1 and 16/1, both at a concentration of 100 nM, as well as a concentration of 200 nM of anti-VEGFA siRNA for the 8/1 ratio (Figure 6a,c,e). As for the control, we used mock siRNA-bearing polyplexes in a similar manner to the proliferation assays. Control polyplexes were formed using x-tremeGene. The data collected showed that mock siRNA/R6p-cRGD polyplexes did not affect the migration of endothelial cells (Figure 6b,d,f). However, when treated with x-tremeGene polyplexes, there was a significant decrease in the migration rate after delivering mock siRNA or anti-VEGFA siRNA (Figure 6h). This finding can be attributed to the inherent cytotoxicity of x-tremeGene polyplexes, which is evident in Figure 3. On the other hand, there is a significant difference between the migration rate of mock and anti-VEGF siRNA-complexes when formed with the R6p-cRGD carrier (Figure 6h). This suggests that the inhibition of endothelial cell migration by the anti-VEGFA siRNA/R6p-cRGD polyplexes is likely due to the RNAi mechanism, which down-regulates the VEGFA gene expression.

In conclusion, it can be stated that, by using anti-VEGFA siRNA/R6p-cRGD polyplexes at optimal charge ratios, we can successfully and specifically inhibit the expression of the VEGFA gene and reduce the proliferation and migration of endothelial cells in vitro. These findings have opened up the possibility for us to explore the therapeutic potential of these polyplexes in an animal EM model.

### 2.6. Assessement of Anti-Angiogenic Properties of siRNA-Complexes In Vivo

The in vivo assessment of the anti-angiogenic effects of delivering anti-VEGFA siRNA by R6p-cRGD-polyplexes was conducted using a surgically induced endometriosis model in rats. The model has been previously established and was utilized to assess the effectiveness of anti-angiogenic therapy [29,48]. The endometrial implants were placed beneath the skin to enable convenient access to the targeted tissue, facilitating the direct injection of the polyplexes to inhibit angiogenesis. Two endometrial fragments were symmetrically implanted in each animal. One implant received injections of either anti-VEGFA or mock siRNA-polyplexes, while the other implant was left untouched. A well-established drug, Dienogest, was used as a positive control for endometriosis treatment, while saline injections served as the negative control. The weight of the animals was monitored during the experiment and no significant changes were detected. The therapeutic potential of the polyplexes was evaluated based on various indicators, including a reduction in the volume of the endometriosis implants, a decrease in VEGFA gene expression, and a decline in the surface expression of CD34, a well-established marker of blood vessels.

**Figure 7:**
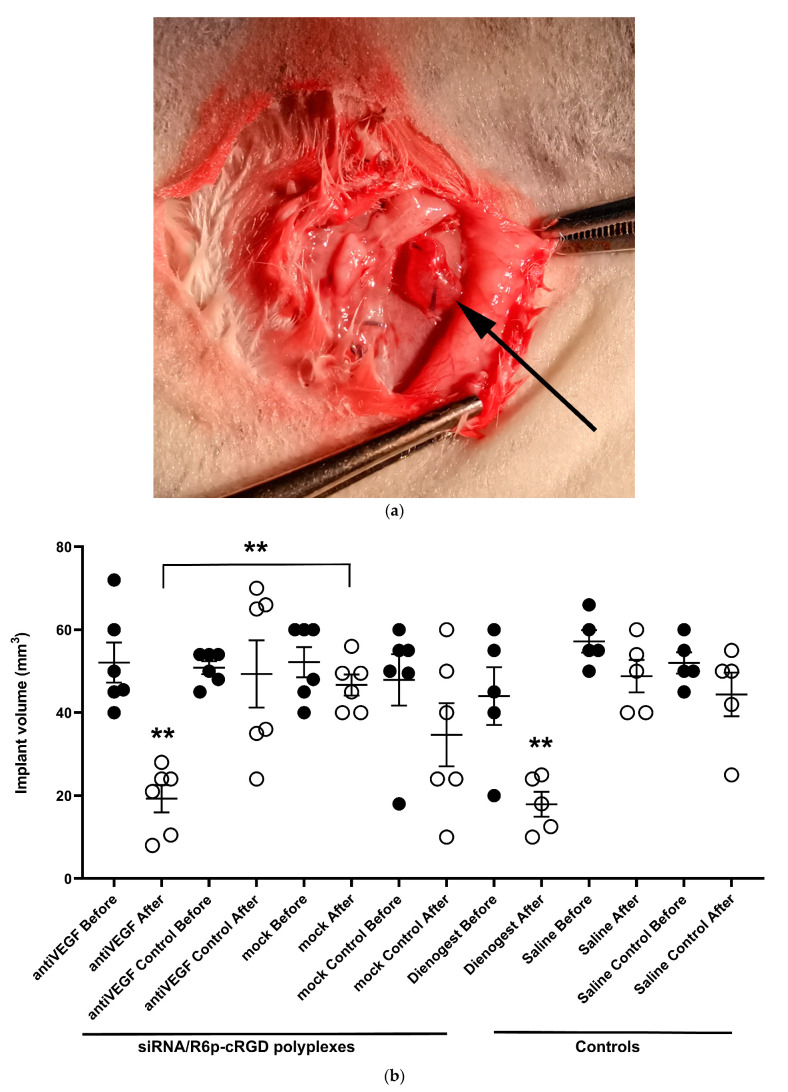
Typical appearance of EM implant (**a**) before injection (indicated by arrow); volume of endometriotic implants (**b**) before and after the treatment with the anti-VEGFA or mock siRNA/R6p-cRGD polyplexes. White dots represent the values after the treatment, while black dots indicate the values prior to it. **—*p* < 0.01 when compared with implants before the treatment. The data are shown as the median ± interquartile range of *n* = 5–6 animals per experimental group; statistical significance was assessed by Kruskal-Wallis test.

The data presented in Figure 7 clearly show that a significant reduction in implant volume was only observed after treatment with anti-VEGFA siRNA-polyplexes and Dienogest. After being treated with anti-VEGFA siRNA-polyplexes and Dienogest, there was a remarkable reduction in the volume of the implants, with a decrease of 2.7-fold and 2.5-fold, respectively. There were no changes in the volumes of control intact implants or implants treated with mock siRNA-polyplexes or saline. The reduction in EM implant volumes was significantly greater in the anti-VEGFA siRNA treatment group compared to the mock siRNA group (*p* < 0.01). These findings lead us to the conclusion that treatment with anti-VEGFA siRNA polyplexes directly contributes to the decrease in EM implant volumes, while the corresponding mock siRNA-polyplexes have no impact on the targeted tissue. Further we investigated the anti-angiogenic effects of the polyplexes by assessing the expression of the VEGFA gene in the implants after siRNA transfection.

**Figure 8:**
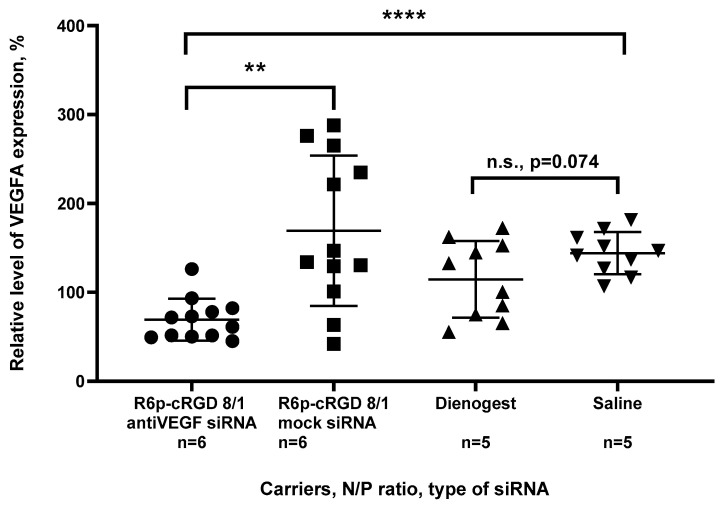
Silencing of VEGFA gene expression in EM implants in vivo after treatment with the siRNA-complexes. The values are given relatively to intact implants. **—*p* < 0.01, ****—*p* < 0.0001 when compared with mock siRNA-polyplexes. The data are shown as the mean ± S.D. of *n* = 5–6 animals per experimental group and two independent measurements per animal; statistical significance was assessed by ordinary one-way ANOVA.

The analysis of VEGFA gene expression in the injected implants revealed a significant two-fold reduction in the target gene expression compared to the control intact implants, but only in the case of anti-VEGFA siRNA-polyplexes (Figure 8). This confirms the specific RNAi-based mechanism of gene silencing, as no significant down regulation was observed after mock siRNA-polyplexes delivery; however, some variability in VEGFA expression can be observed. Interestingly, there was no down regulation of VEGFA after the Dienogest treatment. Although Dienogest has previously been shown to have anti-angiogenic activity, its mechanism of action remains unclear [49]. In various studies on animal models, conflicting data on Dienogest-mediated VEGF gene expression down regulation have been presented [50,51,52]. In our own previous study, we demonstrated a 1.5-fold reduction in VEGFA expression in EM implants after the Dienogest treatment. However, in this current study, no significant changes were observed; however, a tendency can be observed and a further increase in number of animals per experimental group can confirm anti-VEGFA activity of Dienogest. Nevertheless, the obtained results clearly demonstrate significant transfection efficacy of R6p-cRGD-polyplexes in vivo.

**Figure 9:**
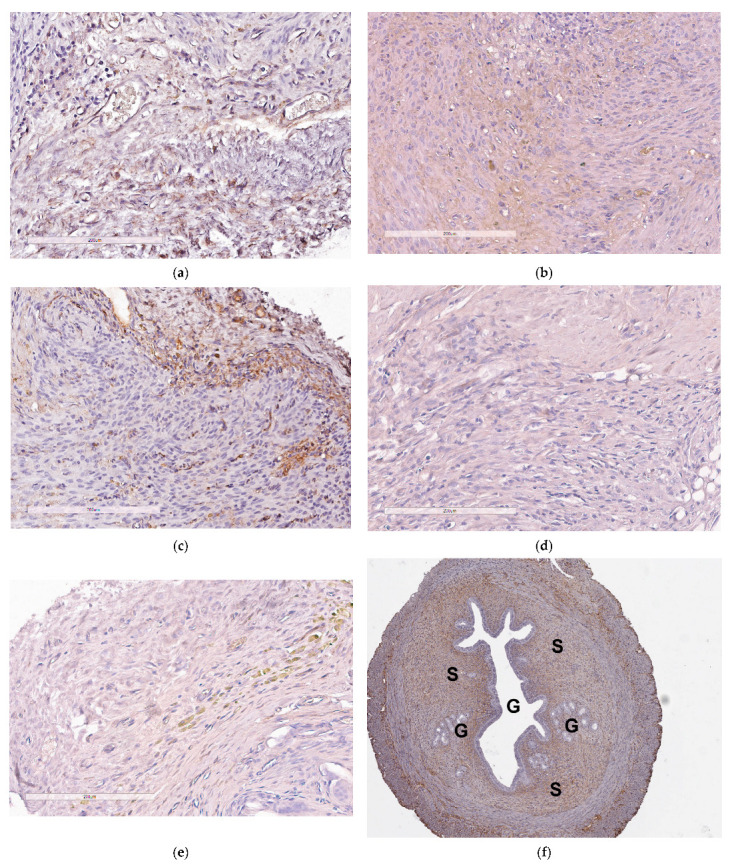
Visual appearance of CD34-stained paraffin sections of endometriotic implants: (**a**) intact contralateral implant; (**b**) implant after the treatment with the mock siRNA-complexes; (**c**) implant after injection of saline; (**d**) implant after the treatment with the anti-VEGFA siRNA-complexes; (**e**) implant after the treatment with Dienogest (magnification 200×, bar represents 200 µm); (**f**) full-size view of intact contralateral EM implant with cyst (G—glandular component; S—stromal component; magnification 40×).

The evaluation of the anti-angiogenic effects produced by the anti-VEGFA siRNA/R6p-cRGD polyplexes was completed by analyzing the CD34 surface expression. The CD34 antigen is found on hematopoietic progenitor cells and endothelial cells, making it a useful marker for differentiating endothelial cells. By utilizing immunostaining for CD34, it becomes possible to detect the development of microvessels that play a vital role in supporting the growth of endometriotic lesions [8]. Due to the limited amount of pathological tissue available, quantitative immunohistochemical analysis was unfortunately not feasible in this study. Nevertheless, Figure 9 reveals that the sections of endometrial implants display distinct staining patterns with CD34. As anticipated, abundant CD34 expression was observed in intact endometriotic implants, as well as in implants after the injection of saline or mock siRNA/R6p-cRGD polyplexes (Figure 9a–c). However, the surface presence of CD34 antigen was not detected following the delivery of anti-VEGFA siRNA (Figure 9d). This significant finding suggests that anti-VEGFA siRNA/R6p-cRGD polyplexes effectively inhibit the growth of microvessels in EM implants. Interestingly, in the Dienogest treated group, some regions with CD34 expression were identified, indicating that a complete inhibition of angiogenesis in EM implants is not present.

The results obtained in our current study can be compared to previous studies that focused on non-viral anti-angiogenic therapy for endometriosis. Previously, it was demonstrated that the delivery of a plasmid encoding pigment epithelium-derived factor using stearic acid-grafted chitosan oligosaccharide micelles led to a significant twofold decrease in the size of endometriotic lesions in rat model of EM. Immunohistochemical analysis showed a marked reduction in microvessel density in the treated rats compared to the control group [53]. Another gene therapy study utilized an endostatin-encoding plasmid complexed with PAMAM dendrimer to demonstrate the anti-angiogenic effect of the treatment. Direct injection of this complex into GFP-expressing subcutaneous endometriotic lesions in nude mice resulted in a 2–3-fold reduction in lesion size compared to the control group over a 30-day period. This finding was supported by a decrease in the expression of the angiogenic factor CD31 [54]. In our efforts to develop an anti-angiogenic approach to endometriosis treatment, we have focused on the down-regulation of VEGFA expression using peptide-based siRNA delivery [29,48]. The R6p-cRGD carrier-mediated delivery we employed demonstrated similar or better anti-angiogenic effects when compared to previously studied approaches. In direct comparison to previously studied RGD1-R6-siRNA polyplexes, it is worth noting that the R6p-cRGD carrier forms more stable and smaller complexes, allowing for a more pronounced reduction in VEGFA protein production. Moreover, in our current study, the anti-VEGFA siRNA/R6p-cRGD polyplexes outperformed Dienogest, which is one of the most effective drugs used for hormonal treatment of endometriosis [55].

## 3. Materials and Methods

### 3.1. Cell Lines and Animals

Both GFP-expressing human triple negative breast cancer cells MDA-MB 231 and human endothelial hybridoma EA.hy926 were cultured without mycoplasma contamination, following the previously described protocols [56,57].

A group of twenty-two female Wistar rats, aged twelve weeks and not pregnant, were used in this study. They were obtained from the Rappolovo Breeding Center (Saint-Petersburg, Russia) and weighed between 180–250 g. The rats were housed in an animal facility and provided with unlimited access to water and a standard diet. Prior to the surgery, the animals were acclimated for 2 weeks. It was necessary for the rats to have a regular menstrual cycle lasting 4–5 days in order to be included in the experimental protocol. The EM surgical modeling followed the guidelines set forth in the Helsinki declaration and the study received approval from the Ethics Committee of the D.O. Ott Research Institute of Obstetrics, Gynecology and Reproductology.

### 3.2. Synthesis of Peptide Carriers

R6 (CHRRRRRRHC), cRGD (cyclic RGDyC(*Npys*)), and cyclo(RGDfK) peptides were synthesized using solid phase Boc-chemistry at NPF Verta, LLC in Saint-Petersburg, Russia (Table 2).

The peptides were stored as a dry powder at an optimal temperature of −20 °C. To ensure their quality, the purity of the peptides was carefully evaluated using HPLC, demonstrating a range of 90–95%. Additionally, the R6p carrier utilized in this study was obtained through a proven methodology as previously described [35]. Briefly, the R6 peptide was dissolved at a concentration of 30 mM with 30% DMSO and subjected to an oxidative polycondensation reaction for 96 h. For R6p-cRGD synthesis, the cRGD moiety was dissolved at the same concentration and added to R6 at a ratio of one cRGD molecule to two R6 molecules before polycondensation. The thiol group of cysteine in the cRGD moiety was shielded using a 3-nitro-2-pyridinesulfenyl group (Npys). This group not only serves as an activating agent for disulfide bond formation, but it is also replaced by the free thiol during the oxidative polycondensation reaction of R6 peptides [58].The resulting R6p and R6p-cRGD were stored in water at a concentration of 2 mg/mL at −70 °C. The number of unreacted thiol groups was determined using Ellman’s assay and expressed as a percentage relative to the absorbance of unpolymerized peptide R6 [35].

### 3.3. siRNAs Sequences

siRNAs were produced at Syntol JSC (Moscow, Russia). The anti-GFP siRNA, with the sense strand sequence of 5′-CAA GCU GAC CCU GAA GUU Ctt-3′, as well as the anti-VEGFA siRNA, with the sense strand sequence of 5′-GCG GAU CAA ACC UCA CCA Att-3′, were previously described in the literature [57]. For control purposes, a mock siRNA, with the sequence of 5′-UUC UCC GAA CGU GUC ACG Utt-3′, was utilized [59].

### 3.4. Preparation of siRNA-Polymplexes

The siRNA/peptide complexes were prepared using different N/P ratios, which refers to the ratio of peptide nitrogen to DNA phosphorus, as previously described [25]. To achieve this, the siRNA was diluted in Hepes-buffered mannitol (HBM), consisting of 5% (*w*/*v*) mannitol and 5 mM Hepes at pH 7.5. The same volume of peptides, corresponding to the desired charge ratio in HBM, was then added to the siRNA solution and vigorously mixed. After that, the complexes were incubated at room temperature for 30 min. As a control, the x-tremeGENE transfection reagent (Roche, Mannheim, Germany) was used in accordance with the instructions provided by the manufacturer.

### 3.5. siRNA Binding Assay

To monitor the binding of peptides to siRNA, we utilized the highly reliable SYBR Green displacement assay, as detailed in a previous study [25]. We employed the d Wallac 1420D scanning multilabel counter (PerkinElmer Wallac Oy, Turku, Finland) to detect the quenching of SYBR Green fluorescence. The binding efficiency was calculated using the formula (F − Ff)/(Fb − Ff), where Ff and Fb represent the fluorescence intensities of SYBR Green in the absence and presence of siRNA, respectively. Notably, the value for unbound siRNA was considered as a reference point of 100%.

### 3.6. RNase A Protection Assay

We prepared peptide/siRNA complexes at different N/P ratios, using a volume of 8 µL as described above. These complexes were then incubated with 100 ng of RNase A (BioChemica AppliChem, Darmstadt, Germany) for 30 min at a temperature of 37 °C. To inactivate the RNase A, we treated the complexes with 1% SDS for 5 min at 98 °C. Afterwards, we exposed the complexes to trypsin (0.1%) at a temperature of 37 °C overnight to release the siRNA. Following this, we electrophoresed the siRNA on a 15% polyacrylamide gel stained with AgNO_3_ [60]. To assess the integrity of the RNA, we compared it with intact siRNA and siRNA treated with RNase A, respectively.

### 3.7. Measurement of Size and Zeta-Potential of siRNA-Complexes

The peptide/siRNA complexes were prepared according to the method mentioned above. Each sample contained 5 µg of siRNA, and the N/P ratios 8/1 and 16/1 were used. The size of the complexes was determined using dynamic light scattering, while the zeta-potential was measured through microelectrophoresis. These measurements were carried out three times using a zetasizer NANO ZS (Malvern Instruments, Malvern, UK).

### 3.8. Cytotoxicity Evaluation of siRNA-Complexes

The cytotoxicity of siRNA/peptide complexes was assessed at N/P ratios of 1/8, 1/16, and 1/24 in MDA-MB-231 and EA.hy926 cell lines. The analysis was conducted in 96-well plates using the Alamar blue assay (BioSources International, San Diego, CA, USA). Cell viability was determined after 16 h of incubation, following a previously described protocol [25]. After transfection the fluorescence of resorufin was measured using a Wallac 1420D scanning multilabel counter with wavelengths of 544/590 nm. The relative fluorescence intensity was calculated as (F − Ff)/(Fb − Ff) × 100%, where Fb and Ff represent the fluorescence intensities in the untreated control and without cells, respectively.

### 3.9. siRNA Transfer to MDA-MB-231 Cells and GFP Fluorescence Detection

The siRNA transfection experiments were conducted in triplicate using MDA-MB-231 cells that had been previously modified to express GFP [25]. A total of 70 × 10^4^ cells were seeded in 48-well plates and left to incubate overnight. The transfections took place in a medium without FBS. Complexes containing anti-GFP siRNA or mock siRNA at concentrations of 200 nM were formed at N/P ratios of 1/8, 1/16, and 1/24 and incubated with the cells for 4 h. After an additional 48 h of incubation in a medium supplemented with FBS, the cells were washed with 1× PBS (pH 7.2) and made permeable using a cell lysis buffer containing 25 mM Gly-Gly, 15 mM MgSO_4_, 4 mM EGTA, 1 mM DTT, and 1 mM PMSF at pH 7.8. The GFP fluorescence was then measured using a Wallac 1420D counter with excitation and emission wavelengths of 485/535 nm. The fluorescence level was normalized by determining the protein concentration in each sample using the Bradford method.

### 3.10. siRNA Transfer to EA.hy926 Cells and Quantitative RT-PCR

Experiments were conducted in triplicate using EA.hy926 cells to silence the VEGFA gene as previously described [29]. We studied the siRNA-complexes at N/P ratios of 1/8 and 1/16, using two different concentrations of siRNA (100 and 200 nM) per well. To analyze the gene expression of VEGFA and β-actin, we employed quantitative real-time PCR with previously reported primers [48]. Each sample was measured three times and the data were averaged to provide a final result. Similarly, we used a comparable protocol to assess VEGFA gene expression in vivo in EM implants. We compared the expression level of VEGFA gene in vivo to the expression level in control animals injected with saline.

### 3.11. Quantitative Measurement of VEGFA Production

EA.hy926 cells were transfected with either anti-VEGFA siRNA or mock siRNA, as explained earlier. Subsequently, we quantitatively assessed the production of VEGFA using a human VEGFA ELISA EH2VEGF kit from Thermo Scientific (Rockford, IL, USA), following the established protocol [29].

### 3.12. Scratch Migration Assay

The migration rate of EA.Hy926 cells was assessed using the method previously described [59]. Briefly, siRNA-complexes were prepared in N/P ratios of 8/1 and 16/1, as explained above. The transfection experiment was carried out in four replicates. The number of cells (n) that migrated to the wound area was then recorded. Additionally, the cell density (ρ) was measured in an area of 17,000 mm^2^. To determine the relative number of migrated cells, the formula (n/n′) × (ρ′/ρ) was used, where n’ represents the number of migrated cells in the untreated control, and ρ’ represents the cell density in the untreated control.

### 3.13. Induction of EM Rat Model and In Vivo siRNA Transfer

We performed surgical modeling of EM based on previously published protocols [29,48,61]. Here, two autologous fragments of the uterus were transplanted onto the outer surface of the abdominal wall in ovariectomized rats. Prior to transfection experiments, the implants were allowed to grow for a period of two weeks. A total of twenty-two rats were randomly divided into four groups (experimental groups have *n* = 6; control groups have *n* = 5). Two more animals were included in the experimental groups to compensate for unexpected adverse effects. Over the course of one week, two injections of either anti-VEGFA siRNA or mock siRNA (10 µg in total) in complexes with R6p-cRGD carrier at an N/P ratio of 1/8 were administered, with a one-week interval between each injection. The rats were anesthetized, and while one endometrial implant received injections of siRNA-complexes at a dose of 5 µg, the contralateral implant was left intact. In the negative control group, endometrial implants were instead injected with an appropriate volume of saline. As for the positive control group, Dienogest (Bayer Schering Pharma AG, Berlin, Germany) in tablet form (crushed in water suspension) was administered orally at a dosage of 1 mg/kg daily for two weeks, following a previously described method [62]. One week after the initial injection, the procedure was repeated, and after an additional week, the rats were sacrificed. Measurements of the injected and contralateral implants’ volumes were taken before and after treatment (L × W × 2 mm^3^), followed by immunohistochemical CD34 detection and VEGFA gene expression analyses, as described previously [29]. Briefly, 3–4 µm thick sections were stained in immunohistostainer BOND-MAX (Leica Biosystems, Wetzlar, Germany) using mouse monoclonal primary antibodies (Abcam UK; CD34 [EP373Y], clone81289). The resulting images (*n* = 4–5 per implant) were obtained using a Leica Aperio AT2 slide scanner and Aperio ImageScope software v.6.25.

### 3.14. Statistical Analysis

Statistical analysis was performed using the GraphPad Prism 8 software v. 8.0.2 package (GraphPad Prism Inc., San Diego, CA, USA). We considered statistical significance for * *p* < 0.05 and ** *p* < 0.01.

## Figures and Tables

**Table 1:** Size and ʐ-potential of the siRNA-complexes.

Carrier	N/P Ratio	Size (nm) ± S.D.	PDI ± S.D.	Z-Potential (mV) ± S.D.
R6p	8/1	140.3 ± 0.5132	0.198 ± 0.013	27.5 ± 1.9
R6p	16/1	150.3 ± 1.1	0.203 ± 0.012	33.2 ± 1.1
R6p-cRGD	8/1	171.8± 1.85	0.211 ± 0.014	23.1 ± 0.5
R6p-cRGD	16/1	188.4 ± 4.1	0.189 ± 0.011	32.1 ± 0.8

**Table 2:** Composition of the polycondensed peptide-based polymers.

	Name	Composition
Monomers	R6	CHRRRRRRHC
	cRGD	C(*Npys*)RGDy |___________|
Polymers	R6p R6p-cRGD	(CHRRRRRRHC)_n_ cRGD-(CHRRRRRRHC)_n_-cRGD

## Data Availability

The data presented in this study are available on request from the corresponding author.
